# Antigen-driven focal inflammatory death of malaria liver stages

**DOI:** 10.3389/fmicb.2015.00047

**Published:** 2015-02-04

**Authors:** Ganchimeg Bayarsaikhan, Masoud Akbari, Katsuyuki Yui, Rogerio Amino

**Affiliations:** ^1^Unité de Biologie et Génétique du Paludisme, Département Parasites et Insectes Vecteurs, Institut Pasteur, Paris, France; ^2^Division of Immunology, Department of Molecular Microbiology and Immunology, Graduate School of Biomedical Sciences, Nagasaki University, Nagasaki, Japan

**Keywords:** CD8^+^ T cells, inflammatory infiltrate, cooperative behavior, *in vivo* imaging, *Plasmodium*

## Abstract

Multiple immunizations using live irradiated sporozoites, the infectious plasmodial stage delivered into the host skin during a mosquito bite, can elicit sterile immunity to malaria. CD8^+^ T cells seem to play an essential role in this protective immunity, since their depletion consistently abolishes sterilizing protection in several experimental models. So far, only a few parasite antigens are known to induce CD8^+^ T cell-dependent protection, but none of them can reach the levels of protection afforded by live attenuated parasites. Systematic attempts to identify novel antigens associated with this efficient cellular protection were so far unsuccessful. In addition, the precise mechanisms involved in the recognition and elimination of parasitized hepatocytes *in vivo* by CD8^+^ T cells still remain obscure. Recently, it has been shown that specific effector CD8^+^ T cells, after recognition of parasitized hepatocytes, recruit specific and non-specific activated CD8^+^ T cells to the site of infection, resulting in the formation of cellular clusters around and in the further elimination of intracellular parasites. The significance of this finding is discussed in the perspective of a general mechanism of antigen-dependent focalized inflammation and its consequences for the elimination of malaria liver stages.

## THE PRE-ERYTHROCYTIC PHASE OF MALARIA INFECTION

Malaria is a mosquito borne disease caused by *Plasmodium spp.* parasites. Despite advances in control and prevention measures, malaria still kills > 600,000 people annually and no effective licensed vaccine is available so far (www.who.int/malaria/en). The disease is a consequence of repeated cycles of parasite invasion and replication inside red blood cells (RBC). However, before infecting the blood and causing the disease, the parasite must pass through a silent and asymptomatic pre-erythrocytic (PE) phase. In mammals, the PE phase starts with the inoculation of sporozoites into the extravascular regions of the host skin during a mosquito bite. Some of these highly motile stages get access to the blood circulation and home to the liver, where they traverse several hepatic cells before invading and developing, as liver stages, inside hepatocytes. One infected hepatocyte generates thousands of RBC-infective stages in 2 to ∼10 days depending on the species. Finally, the PE phase finishes with the release of these invasive stages into the blood circulation (Ménard et al., 2013).

In contrast to the symptomatic erythrocytic phase of infection, which can reach the magnitude of ∼10^12^ circulating infected RBCs in hyperparasitemic adults (World Health Organization [WHO], 1990), the asymptomatic PE stages represent the smallest parasite burden (1∼1000 sporozoites and liver stages) inside the mammalian host (Medica and Sinnis, 2005). Consequently, these stages are considered as ideal targets for vaccine intervention, since early elimination of this minute population of extracellular sporozoites and intracellular liver stages could strategically block infection before pathogenesis and transmission of parasites to mosquitoes. Most importantly, immunizations using live sporozoites, which are blocked during hepatic development as consequence of irradiation (Nussenzweig et al., 1967), genetic modification (Mueller et al., 2005) or drugs (Friesen and Matuschewski, 2011), confer sterile protection against sporozoite re-infection in several experimental models, as well as in humans (Seder et al., 2013).

## THE PUZZLE OF LIVER STAGE KILLING BY CD8^+^T CELLS

Although there is evidence that antibodies and CD4^+^ T cells contribute to the protection induced by live irradiated sporozoites (Schofield et al., 1987; Tsuji et al., 1990; Rodrigues et al., 1993; Doolan and Hoffman, 2000), CD8^+^ T cells seem to be the major players of this sterilizing immunity since in almost all tested rodent (Schofield et al., 1987; Doolan and Hoffman, 2000; Schmidt et al., 2010) and primate (Weiss and Jiang, 2012) models, sterile protection is abolished when CD8^+^ T cells are depleted before sporozoite challenge. Accordingly, the transfer of parasite-specific CD8^+^ T cells can also protect mice from sporozoite infection (Romero et al., 1989; Weiss et al., 1992).

This protective cellular response is associated with a high number of specific CD8^+^ T cells circulating in the peripheral blood of protected mice, ranging from ∼5 to 60% of total circulating CD8^+^ T cells (Van Braeckel-Budimir and Harty, 2014). Similarly, adoptive transfer of ∼10^7^ activated specific CD8^+^ T cells, which totalize ∼26 to 60% of CD8^+^ T cells circulating in the blood, is required to sterilize the infection in the liver, while the transfer of 10^6^ CD8^+^ T cells, which represents ∼3% of CD8^+^ T cells circulating in the blood, is not enough to completely protect mice against sporozoite challenge (Kimura et al., 2013). In addition, interactions between CD8^+^ T cells and infected hepatocytes could not be observed after adoptive transfer of 10^6^ primed CD8^+^ T cells to infected mice (Cabrera et al., 2013), suggesting that the recognition of infected hepatocytes is dependent on high numbers of specific CD8^+^ T cells. In humans, however, the levels of CD8^+^ T cells correlated with protection seem to be much lower than in rodents (Ewer et al., 2013). Protective activity is dependent not only on the quantity, but also on the quality of CD8^+^ T cells. For example, high expression levels of cell adhesion molecules such as CD44 and VLA-4 are correlated with the *in vivo* anti-parasite activity of CD8^+^ T cells, but not with their *in vitro* cytotoxic activity (Rodrigues et al., 1992). These molecules have been implicated in homotypic and heterotypic adhesion of lymphocytes, in the trafficking of T cells to inflamed site (Nandi et al., 2004), and could play an important role in the clustering of T cells at the site of infection (next sections).

Very little is known about the nature of protective parasite epitopes presented on the surface of infected hepatocytes by class I major histocompatibility complex molecules (MHC I). In rodent models, protective MHC I-restricted epitopes were described in the circumsporozoite protein (CSP, Romero et al., 1989; Weiss et al., 1992; Rodrigues et al., 1994; Schmidt et al., 2008) and in the thrombospondin-related anonymous protein (TRAP, Hafalla et al., 2013). Both CSP and TRAP are membrane proteins expressed mainly in sporozoites. In humans, immunization using adjuvanted, truncated CSP induced only partial protection without a detectable specific CD8^+^ T cell response (Moorthy and Ballou, 2009). Immunization using TRAP delivered by viral vectors, which elicit robust T-cell responses, induced also a partial protection which was correlated with the frequency of monofunctional interferon gamma (IFNγ)-producing CD8^+^ T cells (Ewer et al., 2013). Intriguingly, several attempts to identify new epitopes targeted by CD8^+^ T cells among thousands of profiled peptides, mini-genes or genes covering hundreds of parasite proteins, have revealed only a few new PE candidate antigens, but none of them induced protection against sporozoite infection (Mishra et al., 2011; Murphy et al., 2013; Hafalla et al., 2013). Whether the difficulty to unravel new protective epitopes is a consequence of the method of screening, which relies on the secretion of IFNγ by CD8^+^ T cells detected by ELISPOT, or of immunization, which may not reach the critical CD8^+^ T cell threshold necessary for protection, remains to be determined.

Given the scarcity of protective CD8 epitopes, another convenient way to study CD8^+^ T cell responses against infected hepatocytes is to use the epitope of ovalbumin (OVA) fused to endogenous and exogenous antigens expressed in malaria PE stages (Cockburn et al., 2011; Kimura et al., 2013; Montagna et al., 2014). This strategy showed that membrane, cytoplasmic and tubulovesicular network exported proteins can harbor epitopes that are presented on the surface of infected hepatocytes, leading to the parasite elimination by MHC I–restricted (H-2K^b^), OVA-specific CD8^+^ T cells (OT-I cells). Although antigen-presentation is dependent on the transporter associated with antigen processing (TAP; Cockburn et al., 2011; Kimura et al., 2013), which translocates peptides from the cytosol into the endoplasmic reticulum where they are loaded onto MHC I molecules, the mechanism by which these membrane, cytoplasmic and exported parasite antigens cross the parasitophorous vacuole membrane and reach the host cell cytoplasm is still controversial and unclear (Singh et al., 2007; Cockburn et al., 2011; Montagna et al., 2014).

The mechanisms by which CD8^+^ T cells eliminate liver stages are also puzzling. Systemic depletion of IFNγ consistently abolishes the sterile protection induced by irradiated sporozoites in rodent models (Schofield et al., 1987; Doolan and Hoffman, 2000). However, activated IFNγ^–/–^ CD8^+^ T cells harboring transgenic T-cell receptors (TCRs), which can recognize specific cognate epitopes presented on the surface of infected hepatocytes, are still capable of controlling infection as their IFNγ-proficient counterparts (Chakravarty et al., 2008; Kimura et al., 2013). The same is observed for FasL and/or perforin knockout CD8^+^ T cells (Morrot and Zavala, 2004; Kimura et al., 2013). Finally, sterile protection is only lost in a proportion of animals injected with double IFNγ and perforin knockout CD8^+^ T cells (Kimura et al., 2013). Altogether these loss-of-function studies depict a scenario where the elimination of liver stages by specific CD8^+^ T cells seems to be a complex process involving several and redundant effector molecules and probably other IFNγ-producing immune cells.

## CD8^+^ T CELL–DEPENDENT INFLAMMATORY FOCI AND THE DEATH OF LIVER STAGES

While molecular mechanisms underlying the elimination of liver stages by CD8^+^ T cells are still unclear, the cellular events leading to the death of these hepatic parasites are only slowly being revealed. In 1989, multiple inflammatory foci containing numerous CD11b^+^, CD8^+^, and at lesser extent CD4^+^ cells were first observed in the liver of BALB/c mice immunized with *P. berghei* (Pb) irradiated sporozoites, 43 h after challenge with normal sporozoites. Although no parasites could be observed in association with these infiltrates, the formation of these cellular foci was dependent on the presence of CD8^+^ T cells (Hoffman et al., 1989).

Several years later, DNA *in situ* hybridization revealed remnants of *Pb* parasites in close association with cellular infiltrates in the liver of rats immunized with irradiated sporozoites and challenged with normal sporozoites (Scheller et al., 1997). The number of infiltrates increased after 24 h post-challenge and coincided with the decrease in the number of liver stages in the immunized rats. CD4^+^ and CD8^+^ T cells were also identified in these infiltrates. The number of CD4^+^ cells was maintained constant at 31 h and 44 h post-infection, while the number of CD8^+^ T cells was four and sixfold superior to those of CD4^+^ T cells at these respective time points.

Notably, systemic inhibition of nitric oxide (NO) production by aminoguanidine reduced the number of inflammatory foci after 24 h post-infection with a clear diminution of the number of CD8^+^ lymphocytes in the focal infiltrates (Scheller et al., 1997). This treatment also abolished the elimination of liver stages in the immunized animals indicating that the production of NO is critical for the accumulation of CD8^+^ T cells, which are necessary for the parasite elimination. However, the source of inducible NO synthase in the liver of immunized rats challenged with sporozoites seems to be restricted to infected hepatocytes (Klotz et al., 1995). Since NO produced by infected hepatocytes is involved in the elimination of parasites *in vitro* in the absence of T cells (Nussler et al., 1993; Mellouk et al., 1994), it is not possible to distinguish if aminoguanidine abolished elimination of liver stages in the immunized animal by the direct inhibition of NO anti-plasmodial activity, by the inhibition of inflammatory responses that induce the accumulation of CD8^+^ T cells around the infected hepatocytes, or both. Studies using specific CD8^+^ T lymphocytes or liver cells that are deficient in the inducible synthesis of NO could precise the role of this molecule in the killing of liver stages.

## ANTIGEN-DRIVEN INFLAMMATORY FOCI

**FIGURE 1:**
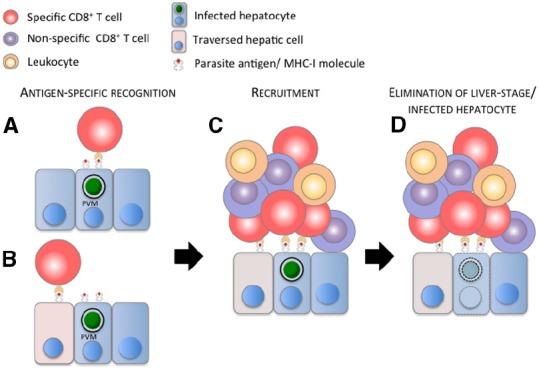
**Model of the elimination of malaria liver stage by a CD8^+^ T cell-dependent inflammatory cell cluster. (A)** Parasite antigens cross the parasitophorous vacuole membrane (PVM), are processed and presented on the surface of the infected hepatocyte by MHC I molecules, and/or **(B)** are released in the cytoplasm and presented on the surface of hepatic cells (hepatocytes, Kupffer cells, endothelial cells—figuratively represented by the pink cells) traversed by sporozoites. **(A,B)** Parasite-specific, activated CD8^+^ T cell (red cell) recognizes its cognate antigen and **(C)** recruits other specific and non-specific (purple cells) CD8^+^ T cells, and presumably other leukocytes (orange cells) to the site of infection. **(D)** These immune cells cluster around the infected hepatocyte, leading to the elimination of the malaria liver stage and presumably of the infected hepatocyte. For clarity and due to the lack of information about its position during cluster formation, the sinusoidal barrier is not represented in the model.

Recently, *in vivo* imaging revealed CD8^+^ T cell infiltrates in close association with infected hepatocytes in BALB/c mice immunized with irradiated *P. yoelii* (Py) sporozoites (Cockburn et al., 2013). The use of fluorescent CD8^+^ T cells harboring transgenic TCRs, specific for epitopes expressed by the parasite and presented by MHC I molecules on the surface of infected hepatocytes, also showed that cluster formation mediated by CD8^+^ T cells is antigen specific. Activated CD8^+^ T cells, which recognize specifically a *Py*CSP-epitope, clustered around and eliminated *Py* infected hepatocytes. Conversely activated OT-I cells did not cluster or eliminate *Py* and *Pb* infected hepatocytes, but readily clustered around and eliminated hepatocytes infected with *Pb* expressing the CD8 epitope of OVA (Cockburn et al., 2013; Kimura et al., 2013). The frequency distribution of *Py*CSP-specific CD8^+^ T cells around infected hepatocytes fitted a mathematical model where the recruitment of T cells was density-dependent, indicating that activated CD8^+^ T cells were attracted to a cluster. The close apposition of CD8^+^ T cells with infected hepatocytes suggests that this first step of specific recognition and recruitment is initiated by the direct contact between these two cells (Figure 1A). Hepatocytes traversed by sporozoites are also thought to present antigens to CD8^+^ T cells. Although traversed hepatocytes do not alter the elimination of infected cells by CD8^+^ T cells *in vitro* (Bongfen et al., 2007), the role of liver cells traversed by sporozoites (Mota et al., 2001; Tavares et al., 2013a,b) located adjacent to infected hepatocytes cannot be formally excluded in the process of initiation and formation of these cellular inflammatory foci (Figure 1B). This capacity of CD8^+^ T cells to recognize a cognate antigen and recruit other CD8^+^ T cells to the site of infection seems to be a general and novel function of CD8^+^ T cells, as reported recently in a model of mouse reproductive tract infection by the lymphocytic choriomeningitis virus (Schenkel et al., 2013).

Surprisingly, when activated *Py*CSP-specific and non-specific CD8^+^ T cells were transferred together in mice infected with *Py* sporozoites, non-specific CD8^+^ T cells, which usually did not cluster around *Py* infected hepatocytes, modified their behavior and were recruited together with *Py*CSP-specific CD8^+^ T cells to the site of infection (Cockburn et al., 2013). In a tumor model, activated OT-I cells were also responsible for the deep infiltration and accumulation of activated non-specific T cells in OVA-expressing EL4 tumors (Boissonnas et al., 2007), indicating that CD8^+^ T cells not only exert a direct cytotoxic activity on target cells, but can also orchestrate a focal inflammatory response by the recognition of a specific antigen and further recruitment of specific and non-specific T cells to the site of infection or tumor growth. This inflammatory role is in agreement with the recent observation that CD8^+^ T cells express a burst of inflammatory cytokines immediately after cognate antigen stimulation recruiting diverse cellular types involved in inflammatory reactions (Sung et al., 2013).

## MULTIPLICITY OF PARASITE DEATH PHENOTYPES

Dynamic imaging revealed how clusters of activated CD8^+^ T cells eliminate liver stages *in vivo* (Cockburn et al., 2013; Kimura et al., 2013). The utilization of a vitality index (VI) based on the fluorescence intensity of GFP-expressing liver stages permitted the monitoring of parasite viability/death over hours of observation after the transfer of activated and specific CD8^+^ T cells. Strikingly, killing of liver stages was a lasting process, which on average occurred after several hours of association between CD8^+^ T cells and infected hepatocytes. In addition, the kinetics of the VI of fluorescent parasites allowed the discrimination of at least three distinct death phenotypes (DPs) associated with the presence of activated CD8^+^ T cells. An abrupt decrease in the parasite VI followed by the diffusion of a weak GFP signal through the host cell cytoplasm characterized the first DP. The second DP was characterized by a slow decrease in the VI over hours of interaction. The blebbing of the infected hepatocyte with the release of parasite material into the sinusoids characterized the third DP. All these distinct DPs suggest again that parasite elimination could rely on multiple and redundant mechanisms of killing.

A better understanding of these killing mechanisms might be achieved using functional *in vivo* imaging. This strategy is a double approach that combines the quantitative imaging of mutant cells with the imaging of non-mutant cells using specific fluorescent reporters to investigate function (Tavares et al., 2013a). For example, the correlation between the ablation of a cytotoxic effector molecule in protective CD8^+^ T cells and the loss of a given DP could indicate a causal relation between these two variables. Complementary, the use of fluorescent markers that report the level of expression of cytotoxic molecules (Croxford and Buch, 2011; Mouchacca et al., 2013) could be used to discriminate different populations of protective CD8^+^ T cells and their relationship with the DPs could be assessed by *in vivo* imaging. The latter approach could also reveal what is the activation phenotype of CD8^+^ T cells associated with efficient homing to the liver, recognition of infected hepatocytes, clustering formation and killing of liver stages.

## CLUSTERING OF CELLS FACILITATES ELIMINATION OF LIVER STAGES

Qualitatively a few CD8^+^ T cells are sufficient to rapidly eliminate a liver stage *in vitro* (Trimnell et al., 2009), however, the killing process *in vivo* seems to be much longer and complex, involving multiple cells (Cockburn et al., 2013). Whether these differences are due to intrinsic characteristics of CD8^+^ T cells used in these studies or are a consequence of limitations imposed by an *in vitro* system is not yet determined. The liver possesses an immune-privileged environment, which confers a relatively high resistance against CD8^+^ T cell responses (Pircher et al., 2006). This fact could explain the long time that these cells require to eliminate liver stages *in vivo*. Cell clustering triggered by antigen-specific CD8^+^ T cells might possibly facilitate the process of parasite killing by augmenting the local concentration of protective cellular and molecular effectors. To test this hypothesis, cluster formation was inhibited by targeting chemokine signaling. Chemokines act on T cells via G-Protein Coupled Receptors (GPCRs) signaling. Pertussis toxin (PTx) was used to inhibit GPCR signaling through the ADP-ribosylation and uncoupling of G-Proteins (Cotton and Claing, 2009). Activated *Py*CSP-specific CD8^+^ T cells treated with PTx had an impaired capacity to cluster around and eliminate infected hepatocytes, while maintaining their capacity to kill target cells and secrete cytokines *in vitro*, as well as, to home to the liver *in vivo* (Cockburn et al., 2013). Although this pharmacological approach indicates that the clustering of cells triggered by *Py*CSP-specific CD8^+^ T cells around infected hepatocytes facilitates the elimination of malaria liver stages, the identification of the chemokine(s) involved in the recruitment of cells to a cluster is still missing. The discovery of these molecules will permit a precise assessment, e.g., using knockout animals, of the role of cell clustering in the elimination of malaria liver stages.

## CONCLUDING REMARKS AND PERSPECTIVES

Altogether these data suggest that CD8^+^ T cell-dependent elimination of liver stages is a complex and cooperative process involving multiple cells and effector molecules. An elevated number of CD8^+^ T cells with a proper binding phenotype seem to be required for finding and eliminating the scarce population of infected hepatocytes in the liver. Following antigen-specific recognition of the site of infection by activated CD8^+^ T cells, both specific and non-specific CD8^+^ T cells and likely other immune cells are recruited to the site of infection. Finally infected hepatocytes are cleared by these antigen-driven inflammatory foci, displaying a multiplicity of DPs. This mechanism of killing places CD8^+^ T cells as an essential linker between a specific adaptive immune response, which starts with the recognition of an infected cell, and a non-specific immune response, characterized by the recruitment of inflammatory cells to the site of infection and ensuing elimination of liver stages (Figure 1). This multi-cellular cooperative model of killing can also incorporate the relative importance of other cellular types, like CD4^+^ T cells and other IFNγ-producing cells, in the foci composition and protection. It may also explain the general dependence on IFNγ for sterile immunity but the dispensable role of this cytokine for protection induced by parasite-specific IFNγ^–/–^ CD8^+^ T cells.

Important topics related to the formation, composition and killing efficacy of these CD8^+^ T cell-dependent inflammatory foci are still unclear and need further investigation. One of these topics is the identification of novel protective CD8 epitopes presented on the surface of hepatocytes (Crabb et al., 2012) and the verification of their concerted action with other known protective epitopes, such as those described in CSP and TRAP. This approach could reveal a possible synergistic, additive, neutral or antagonistic effect on cluster formation and protection. Another important subject to address is the contribution of CD8^+^ T cells, which cannot recognize infected hepatocytes, but are specifically activated in the lymph node that drains the bite site, for example, by mosquito saliva or sporozoite antigens (Chakravarty et al., 2007), in foci formation and elimination of liver stages. Once it is determined that the recruitment of these activated CD8^+^ T cells to infected hepatocytes can improve the killing of liver stages, a vaccination strategy using protective epitopes, presented by infected hepatocytes, and inflammatory epitopes, which might improve killing by focal inflammation, could be envisaged and tested. Similarly the role of non-specific innate immune cells in cluster formation and killing activity is also an open question. It is well documented that inflammatory infiltrates are associated with the natural resistance of naïve rodents to hepatic infection (Khan and Vanderberg, 1991), but their role in immunized animals is not known. In addition, depletion studies using multiple doses of anti-asialo GM1 antibodies partially abrogated protection induced by irradiated sporozoites in several mouse strains (Doolan and Hoffman, 2000). This treatment is known to efficiently deplete NK cells and basophils, and despite the expression of asialo GM1 on subpopulations of NKT, CD8^+^ T and γδ T cells, no significant depletion was observed after a single dose of anti-asialo GM1 antibodies in C57BL/6 mice (Nishikado et al., 2011). This suggests that NK cells, basophils and other innate immune cells could also participate in the focal inflammation orchestrated by specific CD8^+^ T cells. Finally the unraveling of the molecular determinants of highly protective CD8^+^ T cells is crucial for defining the type of effector cells a vaccine should generate. All this information could be very useful for the design of an immunization strategy aiming at the efficient elimination of malaria liver stages mediated by CD8^+^ T cells.

### CONFLICT OF INTEREST STATEMENT

The authors declare that the research was conducted in the absence of any commercial or financial relationships that could be construed as a potential conflict of interest.
